# Integration of multi-omics and clinical treatment data reveals bladder cancer therapeutic vulnerability gene combinations and prognostic risks

**DOI:** 10.3389/fimmu.2023.1301157

**Published:** 2024-01-17

**Authors:** Yan Xu, Xiaoyu Sun, Guangxu Liu, Hongze Li, Meng Yu, Yuyan Zhu

**Affiliations:** ^1^ Department of Urology, The First Hospital of China Medical University, Shenyang, China; ^2^ Department of Pharmacology, School of Pharmacy, China Medical University, Shenyang, China; ^3^ Department of Laboratory Animal Science, China Medical University, Liaoning, Shenyang, China; ^4^ Key Laboratory of Transgenetic Animal Research, China Medical University, Liaoning, Shenyang, China

**Keywords:** bladder cancer, regulation of PD-L1 expression, prognosis, immunotherapy efficacy, molecular docking

## Abstract

**Background:**

Bladder cancer (BCa) is a common malignancy of the urinary tract. Due to the high heterogeneity of BCa, patients have poor prognosis and treatment outcomes. Immunotherapy has changed the clinical treatment landscape for many advanced malignancies, opening new avenues for the precise treatment of malignancies. However, effective predictors and models to guide clinical treatment and predict immunotherapeutic outcomes are still lacking.

**Methods:**

We downloaded BCa sample data from The Cancer Genome Atlas to identify anti-PD-L1 immunotherapy-related genes through an immunotherapy dataset and used machine learning algorithms to build a new PD-L1 multidimensional regulatory index (PMRI) based on these genes. PMRI-related column-line graphs were constructed to provide quantitative tools for clinical practice. We analyzed the clinical characteristics, tumor immune microenvironment, chemotherapy response, and immunotherapy response of patients based on PMRI system. Further, we performed function validation of classical PMRI genes and their correlation with PD-L1 in BCa cells and screening of potential small-molecule drugs targeting PMRI core target proteins through molecular docking.

**Results:**

PMRI, which consists of four anti-PD-L1 immunotherapy-associated genes (*IGF2BP3, P4HB, RAC3*, and *CLK2*), is a reliable predictor of survival in patients with BCa and has been validated using multiple external datasets. We found higher levels of immune cell infiltration and better responses to immunotherapy and cisplatin chemotherapy in the high PMRI group than in the low PMRI group, which can also be used to predict immune efficacy in a variety of solid tumors other than BCa. Knockdown of *IGF2BP3* inhibited BCa cell proliferation and migration, and *IGF2BP3* was positively correlated with PD-L1 expression. We performed molecular docking prediction for each of the core proteins comprising PMRI and identified 16 small-molecule drugs with the highest affinity to the target proteins.

**Conclusions:**

Our PD-L1 multidimensional expression regulation model based on anti-PD-L1 immunotherapy-related genes can accurately assess the prognosis of patients with BCa and identify patient populations that will benefit from immunotherapy, providing a new tool for the clinical management of intermediate and advanced BCa.

## Background

Bladder cancer (BCa) is the tenth most common malignancy worldwide and has the sixth highest incidence in men, with approximately 573,000 new cases and 213,000 cancer-related deaths annually worldwide (1). Patients with muscle-invasive bladder cancer (MIBC) have a poor prognosis, with an overall five-year survival rate of 40–50%. For patients with limited MIBC, neoadjuvant chemotherapy combined with radical cystectomy is the primary treatment modality, whereas only palliative systemic chemotherapy or immunotherapy is used for metastatic BCa (2). BCa is considered an immunotherapy-responsive tumor, immune checkpoint inhibition therapy targeting the immunosuppressive microenvironment has revolutionized cancer treatment (3). However, only a fraction of patients has experienced lasting benefits from immune checkpoint inhibitors, limiting the use of these promising strategies in clinical practice (3, 4). Therefore, there is an urgent need to identify reliable molecular biomarkers to predict the response to checkpoint blockade and improve the clinical efficacy of these therapies.

Research on potential biomarkers for BCa immunotherapy has focused on two aspects, (i) tumor cell-related markers, such as intratumoral heterogeneity (ITH), tumor mutational burden (TMB), intrinsic molecular subtypes (IMS), DNA damage repair (DDR), intrinsic molecular subtypes, gene expression profile (GEP); (ii) tumor microenvironment-related markers, such as tumor-infiltrating lymphocytes (TIL), intrinsic molecular subtypes (IMS), GEP. Damage response (DDR), intrinsic molecular isoforms, GEP. (iii) Markers related to tumor microenvironment, such as tumor infiltrating lymphocyte (tumor-infiltrating lymphocyte TIL), programmed cell death protein (PD-1/PD-L1), gastrointestinal microbiota (5, 6). However, owing to the high heterogeneity of BCa, there is currently no biomarker with sufficient clinical evidence to justify its routine use. Therefore, there is a need to understand the role of immunotherapy in BCa based on immunotherapy-related genes and to identify reliable features to predict the prognosis of patients with BCa and their response to immunotherapy and chemotherapy.

The expression of programmed cell death ligand 1 (PD-L1), an important immunosuppressive protein on the surface of tumor cells, is regulated at multiple levels, including genomic alterations (amplification or translocation), epigenetic modifications (methylation of histones or CpG islands and histone acetylation), transcriptional regulation (inflammatory stimulation and oncogenic signaling), post-transcriptional regulation (miRNA, 3’-UTR, RAS and angiotensin II status regulation), post-translational modifications (ubiquitination, glycosylation, phosphorylation and palmitoylation) (7). The pathways, proteins, and cytokines involved in the regulation of PD-L1 expression are complex and diverse; however, all of these regulatory pathways could be novel ways to treat tumors. In the past few years, ICIs blocking the PD-1/PD-L1 pathway have emerged as therapeutic approaches that can improve the overall survival of patients with mUC, but they are effective with PD-L1 inhibitor therapy only in some patients. Consequently, it is of great clinical significance to investigate the regulatory mechanisms of PD-L1 expression in depth (3). Notably, clinical prediction models constructed to integrate the regulatory mechanisms of PD-L1 expression are lacking. Therefore, exploring PD-L1 expression regulation models can help predict the efficacy of anti-PD-1/PD-L1 immunotherapy in patients with BCa to help them choose the treatment strategies.

In current study,we identified four genes that regulate PD-L1 expression in different dimensions based on the screening of immunotherapy-related genes from an immunotherapy dataset and combined them with machine learning methods to construct a therapeutically guided PD-L1 Multidimensional Regulation Index (PMRI). The different forms of validation all suggest that the PD-L1 multidimensional expression regulation model can accurately assess the prognosis of patients with BCa and identify possible patient populations benefiting from immunotherapy and chemotherapy.

## Materials and methods

### Obtaining sequence data from BCa patients and identifying anti-PD-L1 immunotherapy-related genes

We downloaded expression profile data, corresponding clinical information and pathological sections from The Cancer Genome Atlas (TCGA) (https://portal.gdc.cancer.gov/) database for BCa patients, and raw RNA-seq data were normalized to fragments per kilobase million (FPKM). The GSE13507, GSE32894, GSE31684 and GSE48075 cohorts were obtained from the Gene Expression Omnibus (GEO) (https://cancergenome.nih.gov/) database. We also downloaded the IMvigor210 immunotherapy data cohort, a group of expression profile data and corresponding clinical information for patients treated with anti-PD-L1 antibody (atezolizumab) for uroepithelial carcinoma (8). After receiving anti-PD-L1 antibody immunotherapy, patients were classified into the following four categories based on response: complete remission (CR), partial remission (PR), stable disease (SD), and progressive disease (PD), and we set CR and PR in the IMvigor210 dataset as the group responding to PD-L1 blockers and SD and PD as the group not responding to PD-L1 blockers. Genes differentially expressed in response to anti-PD-L1 immunotherapy were identified by comparing the response and non-response groups by the R package “DESeq2” at a threshold (p-value < 0.05), after which we used WGCNA to obtain the module with the highest correlation to the CR group and thus the anti-PD-L1 immunotherapy-related genes.

### Unsupervised clustering

Based on the red module genes obtained from the above WGCNA, we performed consensus clustering using the R package “ConsensusClusterPlus” to identify BCa subtypes (9).

### Construction and validation of PD-L1 multidimensional regulatory index and histological validation at the protein level

We performed differential expression analysis of the modular genes with the highest correlation with the CR group between tumor and normal tissues with a threshold of logFC > 1 and fdr < 0.01. Genes associated with prognosis in BCa patients were subsequently screened by univariate cox regression analysis with a threshold of p-value < 0.005, and finally multifactorial cox regression analysis was used to construct a PD-L1 multidimensional regulatory index (PMRI). PMRI was obtained for each BCa patient according to the following formula: PD-L1 multidimensional regulatory index (PMRI) = Coef(Gene1) × Expr(Gene1) + Coef(Gene2) × Expr(Gene2) +…… + Coef(Gene n) × Expr(Gene n). Where Expr(Gene n) represents the expression of a particular gene and Coef(Gene n) represents the coefficient obtained from multifactorial Cox analysis of genes. GSE13507, GSE32894, GSE31684 and GSE48075 cohorts were eliminated for batch effects and combined as an external validation cohort.

Based on Kaplan-Meier method and subject operating characteristic curve (ROC) to study TCGA cohort and GSE13507, GSE32894, GSE31684, and GSE48075 cohorts to validate the prognostic value of PMRI. We combined the commonly used clinicopathological features to construct column line plots and compared the validity of the column line plots by plotting 1-, 3-, and 5-year calibration curves as well as ROC curves. We further searched the Human Protein Atlas database (https://www.proteinatlas.org/) (10) to obtain histological validation of *CLK2, IGF2BP3, P4HB* and *RAC3* at the protein level between bladder tumor tissue and normal bladder tissue.

### Gene set enrichment analysis and immuno-infiltration analysis

GSEA was performed by the R package “clusterProfiler” to evaluate the major enrichment pathways in the high-risk group, and the HALLMARK, c5GO and c2KEGG gene sets were set as the enriched gene sets with the screening conditions of |NES| > 1, nominal p-value<0.05. The sample replacement test was performed 1000 times, so gene sets were obtained from the MSigDb database (https://www.gsea-msigdb.org/gsea/msigdb/) download.

Tumor purity, stromal score, immune score and ESTIMATE score (10) were calculated for each BCa patient using the “ESTIMATE” package in the R program. A single sample gene set enrichment analysis (ssGSEA) algorithm was also used to study the level of immune infiltration based on different immune cell types between the high PMRI and low PMRI groups. Lymphocyte scores in pathological sections were graded using a semi-quantitative scoring system (0-5) to describe tumor inflammation.

### Analysis of drug treatment response and immunotherapy response

We derived IC50 by ProPhetic algorithm to assess drug response to common chemotherapy treatments for BCa, comparing drug sensitivity to chemotherapy treatments in patients with high and low PMRI. We also downloaded gene expression of cancer cells to different drugs from Genomics For Drug Sensitivity in Cancer (GDSC) database (https://www.cancerrxgene.org/) (11) and the Cancer Treatment Response Portal (CTRP) database (https://portals.broadinstitute.org/ctrp/) (12) to download the gene expression data of cancer cells to different drugs to analyze the gene expression of *CLK2, IGF2BP3, P4HB* and *RAC3* in relation to drug sensitivity.

The response of BCa patients to immunotherapy was assessed by the Tumor Immune Dysfunction and Exclusion (TIDE) algorithm score in the TIDE database (http://tide.dfci.harvard.edu/) (13), and the immune escape potential between high and low PMRI groups was investigated using the Wilcoxon trial (TIDE score), with high TIDE scores often associated with poorer immunotherapy response and stronger immune escape potential. We also extracted clinical information from the IMvigor210 dataset (atezolizumab) to assess the analysis of differences between anti-PD-L1 immunotherapy groups (responders or non-responders) between high/low PMRI (8). These results were used to assess the predictive value of PMRI on the effect of immune checkpoint therapy.

### Molecular docking simulation

We used MOE software to screen small molecule drugs that bind to the target proteins and perform molecular docking simulations. We downloaded the protein structures of the target targets (CLK2-6KHE, IGF2BP3-6GX6, P4HB-7ZSC and RAC3-2QME) from the PDB database and small molecule drugs were obtained from the zinc15 database of FDA approved drugs and subjected to energy minimization. We set LigX to 7 at pH and 300 K to optimize the protonation state and hydrogen orientation of the protein, and finally simulated the binding pose of IGF2BP3, RAC3, CLK2 and P4HB to small molecule drugs by docking.

### Cell culture

We purchased and used human BCa cell lines T24 and UM-UC3 from the Shanghai Cell Bank of the Chinese Academy of Sciences. T24 and UM-UC3 cells were cultured in medium containing 5% fetal bovine serum against the wall. Cells were cultured at 37°C with 5% CO2.

### Small interfering RNA transfection

The purchased siRNA was transfected with lipo3000. T24 and UM-UC3 cells were pre-plated into 6-well cell culture plates and transfected when cell fusion reached about 70%. First, 7.5 μL of 20 μM siRNA solution and 125 μL of serum-free medium were mixed well and incubated for 5 min at room temperature; 7.5 μL of Lipo3000 and 125 μL of serum-free medium were mixed well and incubated for 5 min at room temperature. The two solutions were then mixed together and incubated for 15 min. Finally, the mixture was added to the cells and the 6-well plate was shaken gently in an “8” motion to mix the transfection solution into the medium. The cells were incubated for 48 hours and then analyzed for transfection efficiency. *IGF2BP3*-specific siRNA sequences are: si-*IGF2BP3*#1, 5’-GCTGGAGCTTCAATTAAGA-3’; si-*IGF2BP3*#2, 5’-CCTTGAAAGTAGCCTATAT-3’.

### Protein blotting

Total cell lysates were extracted, normalized and attenuated for electrophoresis. Proteins were separated by SDS-PAGE and transferred to PVDF membranes. The membranes were then blocked in 3% bovine serum albumin at room temperature, incubated overnight with primary antibody, and then incubated with secondary antibody.

### RNA extraction and real-time PCR

A total of 14 BCa specimens were collected at the First Hospital of China Medical University (Shenyang, China), and all samples were rapidly frozen at -80°C immediately after collection. total RNA was prepared from 14 BCa tissues by Trizol reagent (Invitrogen) and reverse transcribed in PrimeScript RT premix (Takara). The primer sequences for *IGF2BP3* and *PD-L1* primer sequences are shown in **Supplementary Table S1**.

### Cell Counting Kit-8 cell activity assay and EdU cell proliferation assay

CCK8 and EdU were used to detect cell viability. The digested cells were washed and resuspended and then counted using a cell counting plate, and the counts were averaged three times. T24 and UM-UC3 cells transfected with si-*IGF2BP3* were spread in 96-well plates, after which 2000 cells were inoculated into each well, and 6 replicate wells were set up for each group, and five groups of 0h, 24h, 48h, 72h and 96h were set up. At 0h, 24h, 48h, 72h and 96h, 10 μl of CCK8 solution was added to each well and mixed thoroughly with a pipette gun, followed by incubation in a tinfoil-wrapped light-proof treatment in an incubator at 37°C for 4 h. The absorbance values of each well at 450 nm were measured and counted under light-proof conditions using a multi-mode enzyme marker. The si-*IGF2BP3*-transfected T24 and UM-UC3 cells were inoculated in 24-well plates, and each well was incubated with EdU medium for 2 h. The cells were washed twice with PBS. Cell fixation solution (PBS containing 4% paraformaldehyde) was added to each well and fixed at room temperature for 30 min, after which the glycine decolorization shaker was added and incubated for 5 min before discarding the glycine solution and adding 100 μl of permeant decolorization shaker with slow shaking permeation. After that, staining reaction solution was added to each well under light-proof treatment. Images were examined using fluorescence microscopy. Each experiment was repeated three times.

### Cell migration capacity assay

Wound-healing and Transwell assays were performed to determine cell invasion ability. Parallel lines were drawn on the bottom surface of six-well plates using marker pens, T24 and UM-UC3 cells were resuspended and the plates were spread, and the next day a straightedge was used to create scratches than using a 10 μl gun tip with the direction of the scratch perpendicular to the marker line, after which the cells were washed 3 times with PBS to wash away the floating cells, and after 72 h the cells were removed and photographed under a microscope. Transfected T24 and UM-UC3 cells were collected and starved in serum-free medium for 4h to remove the effect of serum. The cells were digested with trypsin and resuspended in serum-free incomplete medium, followed by the addition of the above cell suspension in the upper chamber of the Transwell and complete medium containing 10% fetal bovine serum in the lower chamber for 48 h. The Transwell was washed three times with PBS, and the cells adhering to the lower membrane were fixed with 4% paraformaldehyde and stained with crystal violet. Each experiment was repeated three times.

### Statistical analysis

Survival curves were plotted using the Kaplan-Meier method to compare survival differences between the two groups Correlations were assessed using Spearman correlation analysis. P-values ≤0.05 were considered statistically significant. All statistical analyses were performed by R. Experimental data were processed and plotted using Image J and GraphPad software. The workflow of this study is shown in **Supplementary Figure S1**.

## Results

### Identification of anti-PD-L1 immunotherapy-related genes in BCa

**Figure 1:**
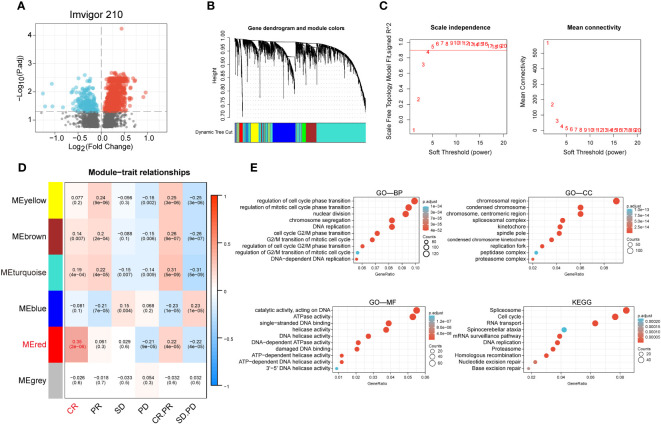
Identification of anti-PD-L1 immunotherapy-related genes in BCa. **(A)** Volcano plot of anti-PD-L1 blocker antibodies (CR and PR groups) versus anti-PD-L1 antibody non-responsive groups (SD and PD groups) for differential analysis. **(B)** Cluster dendrogram of 3301 genes with significant differences. **(C)** Selection of optimal soft threshold power over a wide range. **(D)** Tabular cells showing Pearson correlation coefficients with p-values between modular feature genes and multiple variables. **(E)** GO enrichment analysis and KEGG enrichment analysis of the red module genes.

We performed a difference analysis of the IMvigor210 dataset between the groups responding to PD-L1 blockers (CR and PR groups) and those non-responding to PD-L1 blockers (SD and PD groups) with a threshold of p-value less than 0.05, and obtained 3301 genes and constructed a volcano plot (**Figure 1A**). To identify the modules with the highest correlation with the complete response group (CR group), we applied WGCNA to the TCGA-BLCA dataset to construct a co-expression network and finally aggregated the 3301 genes obtained above into 6 modules (**Figure 1B**) with an optimal soft threshold of 4 to ensure a scale-free topology (**Figure 1C**). The correlations between the module feature genes and multiple variables were obtained by calculating the Pearson correlation coefficient (PCC), where the red module was significantly positively correlated with the CR composition (PCC=0.25, P=2E-06) (**Figure 1D**). We then performed GO and KEGG enrichment analysis of the genes in the red module, and the results showed that they are mainly involved in the cell cycle, DNA replication, and the pathway of DNA unwinding helicase activity (**Figure 1E**).

### Identification of anti-PD-L1 immunotherapy-associated Clusters and differences in immune microenvironment and immunotherapy response among different Clusters

We performed a cluster analysis of BCa patients in the TCGA cohort using the red module genes obtained after WGCNA analysis, and the results showed that the BCa patients in the TCGA cohort could be well divided into two clusters, and there was good internal stability and consistency between the two clusters (**Supplementary Figure S2A**). Kaplan-Meier curves showed that patients in Cluster1 group had significantly worse prognosis than Cluster2 group (p < 0.05) (**Supplementary Figure S2B**). Comparison of the differences in immune microenvironment between the two Clusters by the ESITIMATE algorithm showed that Cluster 2 had a higher ESITIMATE score, immune score, stromal score and lower tumor purity compared to Cluster 1 (**Supplementary Figure S2C**). The CIBERSORT algorithm showed a significant difference in immune cell infiltration between the two Clusters. Significantly different, with a significantly higher proportion of CD8 T cells in Cluster 2 than in Cluster 1 (p < 0.05) (**Supplementary Figure S2D**).

### Construction and validation of PD-L1 multidimensional regulatory index

**Figure 2:**
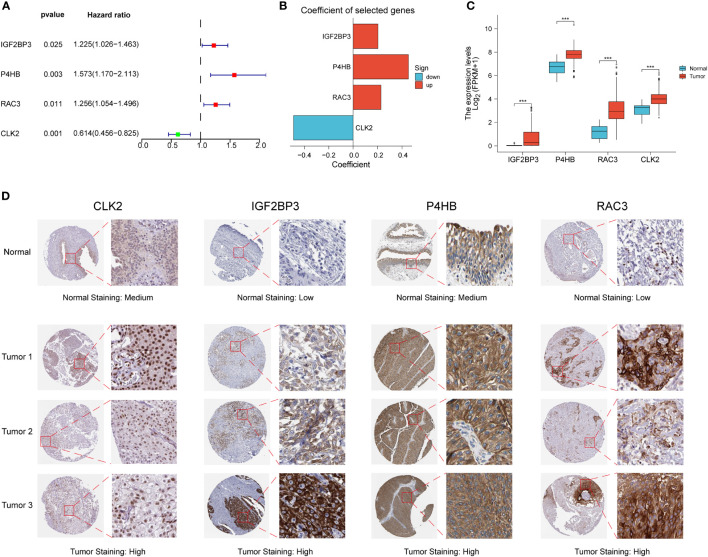
Construction of PD-L1 multidimensional regulatory index and validation of immunohistochemical staining results. **(A)** Forest plots of four PMRIs obtained by multifactorial Cox analysis. **(B)** Constructed histograms of four gene coefficients composed of PMRI. **(C)** Comparison of the expression levels of *IGF2BP3, P4HB, RAC3* and *CLK2* in TCGA BCa tissues and normal tissues. **(D)** Immunohistochemical staining results of CLK2, IGF2BP3, P4HB and RAC3 in normal and BCa tissues. *p < 0.05, **p < 0.01, ***p < 0.001.

We performed differential and prognostic analyses on the red module genes obtained from the WGCNA analysis, after which in order to construct a risk model associated with PD-L1 immunotherapy and its derived PD-L1 multidimensional regulatory index (PMRI), we used multifactorial Cox analysis to screen four genes with independent prognostic value to construct the PMRI, which were *IGF2BP3* (HR= 1.225, 95% CI=1.026-1.463, P=0.025), *P4HB* (HR=1.573, 95% CI=1.170-2.113, P=0.003), *RAC3* (HR=1.256, 95% CI=1.054-1.496, P=0.011) and *CLK2* (HR=0.614, 95% CI=0.456-0.825, P=0.001)(The coefficients obtained from the multifactorial Cox analysis of the four genes in PMRI were 0.203 for *IGF2BP3*, 0.453 for *P4HB*, 0.228 for *RAC3* and -0.488 for *CLK2* (**Figures 2A, B**). We compared the expression levels of *IGF2BP3, P4HB, RAC3* and *CLK2* in TCGA BCa tissues and normal tissues, and the results showed that all four genes were significantly upregulated in tumor tissues (**Figure 2C**). We also obtained immunohistochemical staining results of *CLK2, IGF2BP3, P4HB* and *RAC3* from the HPA database in normal and BCa tissues, demonstrating that these four genes were highly expressed in BCa tissues (**Figure 2D**).

### PD-L1 multidimensional modulation index predicts the prognosis of BCa patients

**Figure 3:**
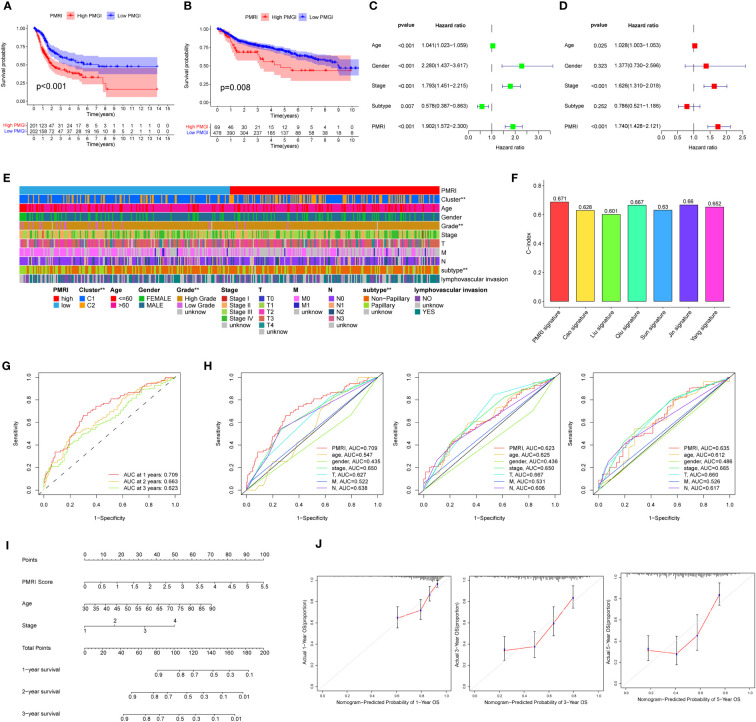
Association of PD-L1 multidimensional regulatory indices with clinical traits and construction of line plots. **(A)** Kaplan-Meier survival curves for the TCGA cohort. **(B)** Kaplan-Meier survival curves for the GSE13507, GSE32894, GSE31684 and GSE48075 cohorts. **(C, D)** Univariate and multivariate regression analyses. **(E)** Common clinical characteristics (Cluster, age, sex, grade, stage, T, M, N, BCa subtype and whether lymphovascular invasion) were analyzed for differences in the high/low PMRI group. **(F)** Histogram of PMRI index compared with other BCa prognostic indices. **(G)** ROC curves of PMRI at 1, 3 and 5 years. **(H)** AUC comparison of PMRI with other clinical traits at 1, 3 and 5 years. **(I)** Column line graphs constructed based on PMRI, age, and clinical stage. **(J)** Calibration curves for 1-year, 3-year and 5-year overall survival. *p < 0.05, **p < 0.01, ***p < 0.001.

Kaplan-Meier curves showed that the high PMRI group had a worse prognosis in the OS phase compared to the low PMRI group (p < 0.05) (**Figure 3A**). To assess the prognostic predictive validity of PMRI, we obtained the GSE13507, GSE32894, GSE31684, and GSE48075 cohorts as a validation cohort, and the results showed that the mortality rate was significantly higher in the high-PMRI group than in the low-PMRI group (p < 0.05), suggesting that PMRI has a better prognostic predictive value at OS stage (**Figure 3B**). The results by univariate and multivariate regression analysis showed that PMRI was an independent risk factor (**Figures 3C, D**). We also performed a difference analysis of different common clinical characteristics in the high/low PMRI group, and the results showed that Cluster, grading and BCa subtypes were significantly different in the high/low PMRI group (p < 0.05) (**Figure 3E**). Analysis of PMRI index and clinical trait stratified survival curves showed that PMRI could significantly differentiate the prognosis of each clinical subgroup, with patients in the high PMRI group having a had a poorer prognosis (**Supplementary Figure S3**).

We also obtained the characteristics of currently published prognostic models for BCa and compared them with the prognostic prediction accuracy of the PMRI in this study, and the results showed that the PMRI outperformed other prognostic models in predicting BCa patients (**Figure 3F**; **Supplementary Figure S4**), (14–20).The area under the ROC curve (AUC) can be used to analyze the validity of the PMRI prognostic prediction, and the AUCs at 1, 3 and 5 years were 0.709, 0.663 and 0.623, and the 1-year AUC was better than other clinical features in predicting patient survival, suggesting that this PMRI can better predict the short- and long-term survival of BCa patients (**Figures 3G, H**). Finally, we constructed column line graphs based on the PMRI and other clinical features (age and clinical stage) from multivariate analysis, with the PMRI accounting for the major part of the total column line graph score (**Figure 3I**). The calibration curve showed that the 1-, 3-, and 5-year column line plots exhibited good predictive accuracy compared to the reference line (**Figure 3J**). These results above suggest that PMRI can accurately and reliably predict the survival outcome of BCa patients.

### PD-L1 multidimensional regulatory index predicts pan-cancer prognosis

**Figure 4:**
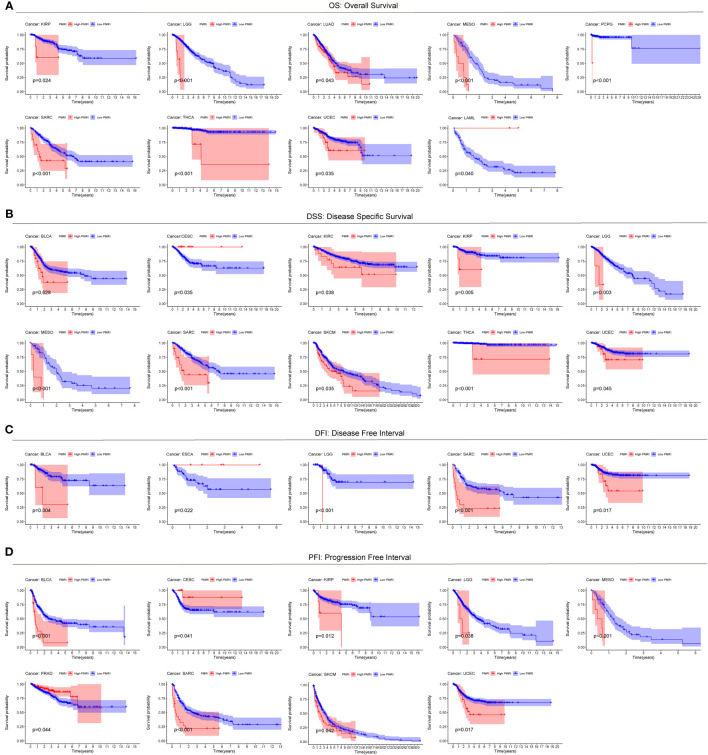
Predictive value of PMRI in other cancers. Using Kaplan-Meier survival curve analysis, patients in the high PMRI group and low PMRI group were compared in pan-cancer for Overall Survival (OS) **(A)**, Disease Specific Survival (DSS) **(B)**, Disease Free Interval (DFI) **(C)**, and Progression Free Interval (PFI) **(D)**.

To explore the prevalence of PMRI in other cancers, we used the model equation for PMRI described above to calculate PMRI values for patients with other cancer types in TCGA and to plot Kaplan-Meier survival curves for the high/low PMRI groups. For Overall Survival (OS), patients in the high PMRI group in KIRP, LGG, LUAD, MESO, PCPG, SARC, THCA, and UCEC had a poorer prognosis, while patients in the low PMRI group in LAML had a poorer prognosis (**Figure 4A**). For Disease Specific Survival (DSS), patients in the high PMRI group in BLCA, KIRC, KIRP, LGG, MESO, SARC, SKCM, THCA, and UCEC had poorer Disease Specific Survival. The patients in the low PMRI group in CESC had poorer Disease-Specific Survival (**Figure 4B**). For Disease Free Interval (DFI), patients in the high PMRI group in BLCA, LGG, SARC and UCEC had shorter Disease Free Interval, while patients in the low PMRI group in ESCA had shorter Disease Free Interval (**Figure 4C**). For Progression Free Interval (PFI), patients in the high PMRI group had shorter Progression Free Interval in BLCA, KIRP, LGG, MESO, SARC, SKCM and UCEC, while patients in the low PMRI group had shorter Progression Free Interval in CESC and PRAD. Progression Free Interval in CESC and PRAD (**Figure 4D**). The above results suggest that PMRI has a good effect in predicting prognosis not only in BCa but also in other cancers.

### Correlation of gene set enrichment analysis and PD-L1 multidimensional regulatory index with the tumor microenvironment

**Figure 5:**
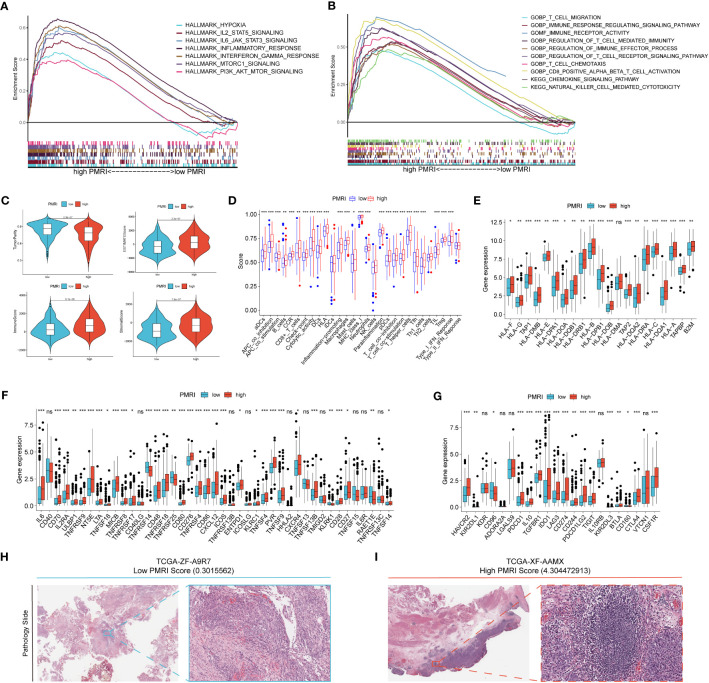
Correlation analysis of GSEA and tumor microenvironment. **(A, B)** GSEA analysis of patients in the high PMRI group. **(C)** Comparison of tumor purity, ESTIMATE score, immune score and stromal score between patients in the high PMRI group and low PMRI group. **(D)** Comparison of immune-related functions between patients in the high PMRI group and low PMRI group. **(D)** Comparison of HLA molecule **(E)**, immunostimulatory gene **(F)** and immunosuppressive gene **(G)** expression between patients in the high PMRI group and low PMRI group. Level of immune cell infiltration in TCGA pathological sections of patients in the low PMRI group **(H)** and patients in the high PMRI group **(I)**. *p < 0.05, **p < 0.01, ***p < 0.001, ns, Non Significance.

To explore the cancer signaling pathways associated with PMRI, we performed GSEA analysis in the high PMRI and low PMRI groups, and the results showed that the high PMRI group was significantly enriched in HYPOXIA, IL2_STAT5_SIGNALING, IL6_JAK_STAT3_SIGNALING, INFLAMMATORY_ RESPONSE, INTERFERON_GAMMA_RESPONSE, MTORC1_SIGNALING, and PI3K_AKT_MTOR_SIGNALING signaling pathways, and the above signaling pathways are involved in the regulation of PD-L1 expression levels, so it can be confirmed that PMRI is closely related to PD-L1 expression levels (21) (**Figure 5A**). In addition, by performing GO and KEGG enrichment analysis on the high PMRI group, the results showed that high PMRI was closely associated with immune cell infiltration (**Figure 5B**).

We used the ESTIMATE algorithm to assess immune cell infiltration in the tumor microenvironment of BCa patients, and the results showed that patients in the high PMRI group had significantly higher TME scores (ESTIMATE score, immune score and stromal score) and significantly lower tumor purity scores than those in the low PMRI group, indicating that compared to the low PMRI group, patients in the high PMRI group had a higher percentage of immune and stromal cell infiltration ratio was higher and tumor purity was lower in the high PMRI group compared to the low PMRI group (**Figure 5C**). The results of immune-related function analysis by ssGSEA algorithm showed that HLA and immune checkpoint expression were significantly higher in the high PMRI group than in the low PMRI group (**Figure 5D**). We then compared the expression of common HLA molecules, immunostimulatory genes and immunosuppressive genes in the high/low PMRI group, and most common HLA molecules, immunostimulatory genes and immunosuppressive genes were highly expressed in the high PMRI group, such as PD-L1, PD-1 and CTLA-4 (**Figures 5E–G**). We confirmed the higher level of immune cell infiltration in patients in the high PMRI group (TCGA-XF-AAMX) than in patients in the low PMRI group (TCGA-ZF-A9R7) by TCGA pathological sections (**Figures 5H, I**). Taken together, the results suggest that our patients in the high PMRI group may correspond to BCa hot tumors while those in the low PMRI group may correspond to BCa cold tumors.

### Association of PD-L1 multidimensional modulation index with immunotherapy efficacy

**Figure 6:**
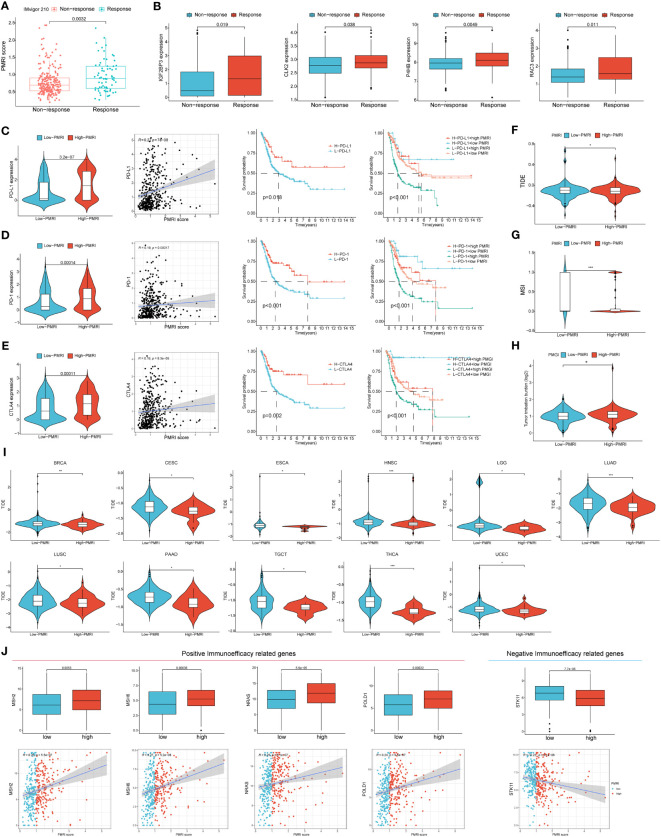
PD-L1 multidimensional modulation index in response to immunotherapy. **(A)** Box plots of PMRI scores in the responding and non-responding groups. **(B)** Differences in *IGF2BP3, CLK2, P4HB* and *RAC3* expressions between the responding and non-responding groups. **(C–E)** Differences in *PD-1, PD-L1* and *CTLA-4* expression between high/low PMRI groups, correlation of PMRI with PD-1, PD-L1 and CTLA-4 expression and prognostic correlation of PMRI with *PD-1, PD-L1* and *CTLA-4*. Differences in TIDE score **(F)**, MSI **(G)** and TMB **(H)** between the high PMRI group and low PMRI group. **(I)** PMRI scores are used to assess immunotherapy efficacy in pan-cancer. **(J)** Association of PMRI index with genes related to positive/negative immune efficacy. *p < 0.05, **p < 0.01, ***p < 0.001.

We used the IMvigor 210 database to analyze the immunotherapy response in the high/low PMRI population, and the results showed that the PMRI scores of those who responded to anti-PD-L1 antibodies were significantly higher than those who did not respond to anti-PD-L1 antibodies, indicating that high PMRI scores were strongly associated with better immunotherapy outcomes (**Figure 6A**). We also determined the difference in expression of the four genes that construct the PMRI score between the responding and non-responding groups, and the results showed that all four genes (*IGF2BP3, CLK2, P4HB* and *RAC3*) were significantly more highly expressed in those who responded to anti-PD-L1 antibodies (**Figure 6B**). In addition, higher expression levels of the immune checkpoints *PD-1, PD-L1* and *CTLA-4* in the high PMRI group and a significant positive correlation between PMRI score and expression of *PD-1, PD-L1* and *CTLA-4* were strongly associated with a good prognosis in the OS phase of BCa (**Figures 6C–E**). Patients with lower TIDE scores were more likely to benefit from immunotherapy (13) and high PMRI group had a significantly higher TIDE score than the low PMRI group, and it can be inferred that the high PMRI group responded better to immunotherapy (**Figure 6F**). In addition, we found significant differences in MSI and TMB between the high/low PMRI groups (**Figures 6G, H**). Finally, we assessed the value of PMRI score in predicting the efficacy of immunotherapy by PMRI scoring in cancer patients in pan-cancer, and the results showed significant differences in TIDE scoring between the low PMRI scoring group and the high PMRI group in BRCA, CSC, ESCA, HNSC, LGG, LUAD, LUSC, PAAD, TGCT, THCA, and UCEC, suggesting that PMRI scores can also be used to assess immunotherapy efficacy in pan-cancer in addition to in BLCA (**Figure 6I**). In addition, we collected genes that have been currently reported to be positively associated with immune efficacy and negatively associated with immune efficacy and analyzed them for association with PMRI (22–24). The results showed that PMRI was significantly positively associated with positive immune efficacy-related genes (*MSH2, MSH6, NRAS* and *POLD1*) and negatively associated with negative immune efficacy-related genes (*STK11*) (**Figure 6J**). The above results suggest that PMRI can predict the effect of immunotherapy and that patients in the high PMRI group have a better effect on immunotherapy.

### Association between PD-L1 multidimensional modulation index and chemotherapy response and common drug sensitivity

To guide the clinical use of drugs in BCa patients, we analyzed the response to common BCa chemotherapeutic drugs in the high PMRI and PMRI groups, and the IC50 was negatively correlated with patients’ sensitivity to the drugs. The results showed that patients in the high PMRI group were more effective in treatment with Cisplatin, while the low PMRI group was more effective in treatment with Gefitinib and Methotrexate treatment was more effective (**Supplementary Figure S5A**). We collected data from the TCGA cohort to analyze the difference in PMRI scores between the cisplatin-responsive group and the non-responsive group. The results showed that the PMRI score in the cisplatin-responsive group (CR&PR) was significantly higher than that in the cisplatin-non-responsive group (PD&SD) (**Supplementary Figure S5B**). In addition, the relationship between drug sensitivity and mRNA expression of *P4HB, IGF2BP3, RAC3* and *CLK2* was analyzed by GDSC and CTRP databases, with positive correlation representing gene expression associated with drug resistance and negative correlation representing genes associated with drug sensitivity. The results of both databases showed that the mRNA expression of *P4HB* correlated with most chemotherapeutic drug resistance, and the mRNA expression of *CLK2* correlated with most chemotherapeutic drug sensitivity (**Supplementary Figures S5C, D**). These results suggest that PMRI is an effective indicator for predicting the efficacy of commonly used chemotherapy drugs (cisplatin and methotrexate) in BCa, and can be used as a potential therapeutic target for BCa chemotherapy.

### Small molecule drug candidate prediction for core target proteins

**Figure 7:**
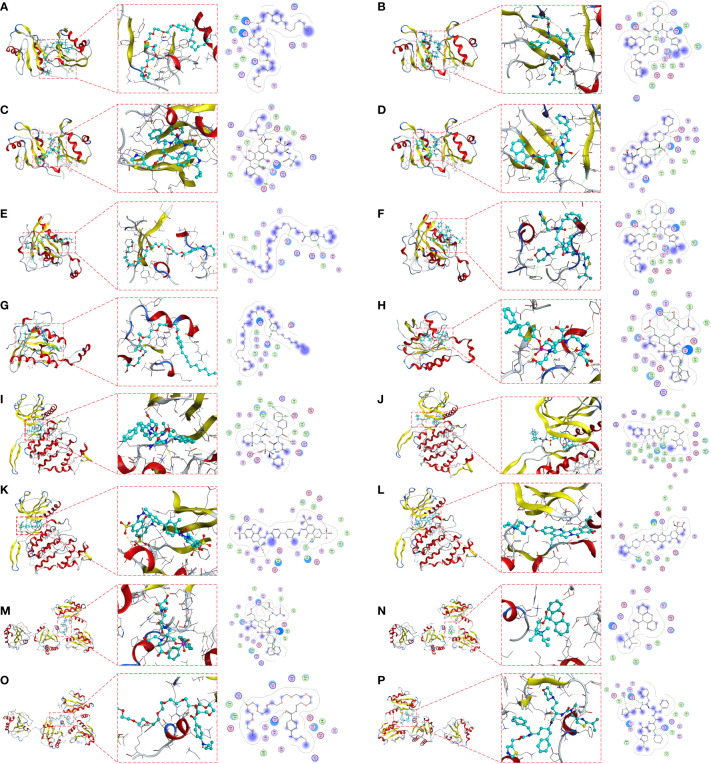
Molecular docking pose. Screening of candidate small molecules for target proteins using molecular docking. Docking poses of IGF2BP3 active pocket with Nonoxynol-9 **(A)**, Cobicistat **(B)**, Valrubicin **(C)** and Indinavir **(D)** are shown. Docking poses of RAC3 active pocket with Tessalon **(E)**, Cobicistat **(F)**, Nonoxynol-9 **(G)** and Docking poses of the CLK2 active pocket with Saquinavir **(I)**, Amaryl **(J)**, Trypan Blue **(K)** and Irinotecan **(L)**. Docking poses of the P4HB active pocket with Gadofosveset **(M)**, Propantheline **(N)**, Tessalon **(O)** and Cobicistat **(P)** in docked position. On the left is the overall structure of IGF2BP3, RAC3, CLK2 and P4HB with small molecule drugs, where the small molecules are embedded into the protein. In the middle is a detailed diagram of the interaction between IGF2BP3, RAC3, CLK2 and P4HB with small molecule drugs, where the hydrogen bonds are shown as yellow dashed lines. On the right is a 2D interaction diagram between IGF2BP3, RAC3, CLK2 and P4HB and small molecule drugs, where green arrows indicate side chain hydrogen bonding interactions, blue arrows indicate main chain hydrogen bonding interactions, and hexagons represent residues interacting with aromatic hydrocarbons.

Molecular docking is a structure-based computational algorithm for compound screening. We obtained the protein structures of IGF2BP3, RAC3, CLK2 and P4HB from the PDB database for molecular docking with 1379 FDA-approved small molecule drugs. Showing the top four small molecules with the highest binding power to the IGF2BP3 binding pocket (Nonoxynol-9, Cobicistat, Valrubicin, and Indinavir) (**Figures 7A–D**), the top four small molecules with the highest binding power to the RAC3 binding pocket (Tessalon, Cobicistat, Nonoxynol-9, and Gadofosveset) (**Figures 7E–H**), the top four small molecules with the highest pocket binding to CLK2 (Saquinavir, Amaryl, Trypan Blue, and Irinotecan) (**Figures 7I–L**), and the top four small molecules with the highest pocket binding to P4HB The top four small molecules with the highest pocket binding (Gadofosveset, Propantheline, Tessalon and Cobicistat) (**Figures 7M–P**). For example, Cobicistat (ZINC000085537014) forms hydrogen bonds with IGF2BP3 amino acid residues Glu-69, Ser-58 and Arg-79, where Glu-69 and Ser-58 act as hydrogen bond acceptors and Arg-79 as a hydrogen bond donor. In addition, these small molecules form van der Waals (VDW) interactions with residues around the protein receptor, contributing to the binding between the small molecules and IGF2BP3, RAC3, CLK2 and P4HB.

### Knockdown of *IGF2BP3* inhibits proliferation and migration of BCa and *IGF2BP3* positively correlates with PD-L1

**Figure 8:**
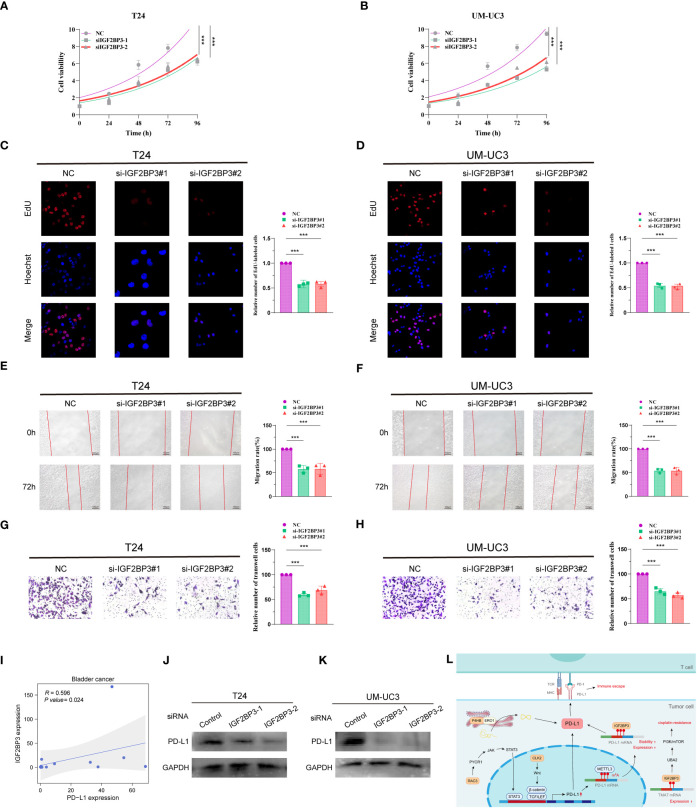
Knockdown of *IGF2BP3* inhibits BCa cell proliferation and migration and *IGF2BP3* correlates with PD-L1. CCK8 viability assay **(A, B)**, EdU cell proliferation capacity assay **(C, D)**, wound healing capacity assay **(E, F)** and transwell cell migration capacity **(G, H)** in T24 and UM-UC3 cells transfected with two si-*IGF2BP3*, respectively. **(I)** qPCR assay of *IGF2BP3* and *PD-L1* correlation in 14 BCa tissues. **(J, K)** PD-L1 protein expression levels in T24 and UM-UC3 cells after siRNA transfection. **(L)** PMRI index gene and PD-L1 correlation pattern plot. *p < 0.05, **p < 0.01, ***p < 0.001.

To investigate the role of *IGF2BP3* in BCa cells, two siRNAs (*IGF2BP3*-1, *IGF2BP3*-2) were designed to silence *IGF2BP3* expression in T24 and UM-UC3 cells. The expression of IGF2BP3 after knockdown was verified by protein blotting, and the results showed that the above two siRNAs could effectively knock down the expression of *IGF2BP3* (**Supplementary Figures S6A, B**). We then performed CCK8, EdU, wound healing and Transwell experiments on T24 and UM-UC3 cells transfected with si-*IGF2BP3*, respectively. The CCK8 results showed that the proliferation ability of T24 and UM-UC3 cells in the NC group was significantly higher than that of both si-*IGF2BP3* groups at 24, 48, 72 and 96 h (p < 0.05, **Figures 8A, B**). The results by EdU staining assay showed that knockdown of *IGF2BP3* gene had significantly lower proliferative capacity for T24 and UM-UC3 cells than the NC group (p < 0.05, **Figures 8C, D**). Both wound healing assay and Transwell assay results showed that the migratory ability of T24 and UM-UC3 cells with low expression of *IGF2BP3* was significantly reduced (p < 0.05, **Figures 8E–H**). The above results suggest that knockdown of *IGF2BP3* expression can inhibit the proliferation and migration of BCa cells.

We then collected 14 BCa tissue samples to investigate the correlation between IGF2BP3 and *PD-L1*, and found a significant positive correlation between *IGF2BP3* and *PD-L1* (R=0.596, P value=0.024, **Figure 8I**). We found a significant decrease in the protein level of *PD-L1* after silencing *IGF2BP3* expression in T24 and UM-UC3 cells by WB experiments (**Figures 8J, K**; **Supplementary Figures S6C, D**). In addition, we constructed a pattern diagram of the relationship between the four genes constructing the PMRI index and PD-L1 (**Figure 8L**).

## Discussion

Advanced or metastatic BCa is an aggressive malignancy associated with poor long-term survival outcomes (17, 25, 26). Immune checkpoint inhibitors targeting programmed cell death 1 (PD-1) or PD-L1 have recently been shown to be effective in patients with BCa (27). Therefore, more accurate and reliable immunotherapy-related markers are needed to predict survival status and immunotherapy response in patients with BCa. In this study, we established a PMRI constructed from four anti-PD-L1 immunotherapy-related genes (*IGF2BP3, P4HB, RAC3*, and *CLK2*) based on an anti-PD-L1 immunotherapy dataset. We found an association between high PMRI not only with poor prognosis in patients with BCa, but also with therapeutic strategies that could help differentiate and predict the efficacy of immunotherapy in patients with BCa, and thus improve patient prognosis. *In vitro* experiments and preliminary virtual screening further identified the biological functions and potential druggability of PMRI. Thus, our PMRI can predict the prognostic risk and immunotherapy response of BCa. The derived PMRI can be used as a new indicator to predict BCa prognosis and immunotherapy benefits, and may provide valuable insights to find new BCa treatment strategies.

An important finding of this study was the exploration of the therapeutic potential for BCa from the new perspective of PD-L1 expression co-regulation patterns. To determine whether PMRI can predict the efficacy of anticancer immunotherapy in patients with BCa, we compared the expression levels of common immune checkpoints and HLA molecules between the high and low PMRI groups and found that the majority were significantly upregulated in the high PMRI group, with *PD-1, PD-L1*, and *CTLA-4* expression levels significantly correlated with PMRI scores. The results showed that the TIDE scores in the high PMRI group were significantly lower than those in the low PMRI group, suggesting that ICI immunotherapy is more effective in treating the high PMRI population. We also scored PMRI for TIDE immune efficacy in pan-cancer, demonstrating that it can be used not only as a potential biomarker to predict BCa immunotherapy response and screen suitable patients, but also for predicting immunotherapy efficiency in pan-cancer. Consistent with the TIDE score results, there were significant differences in PMRI scores between the responding and non-responding groups of uroepithelial cancer patients in response to PD-L1 therapy in the IMvigor 210 cohort, with the responding group having higher PMRI scores, validating the efficacy of anti-PD-L1 immunotherapy for patients in the high PMRI score group. However, we predicted the response of BCa patients to treatment with common chemotherapeutic agents based on the IC50 values of the drugs. Additionally, we calculated the association between gene expression and drug sensitivity, and found that common chemotherapeutic agents (e.g., cisplatin and gemcitabine) also differed significantly between PMRI subgroups. These results suggest that we constructed the PMRI as a valid indicator to assess the response of patients with BCa to immunotherapy and chemotherapy, to accurately assess the prognosis of patients, and to identify the patient population that will benefit from immunotherapy.

Current predictive biomarkers for PD-1/PD-L1 inhibitors include predictors, such as TMB, MSI, PD-L1, IFN-γ, TIL, and serum markers (IL-6/IL-8/LDH/CRP/B2M) (5). The other cytokines and serum markers predict the efficacy of immunotherapy in terms of the immune microenvironment. In our study, we constructed a PMRI based on immunotherapy datasets and from the perspective of the multifaceted regulation of *PD-L1* expression in cancer cells and found that it not only correlated significantly with TMB and MSI, but also validated its effectiveness in predicting immunotherapy efficacy in patients with BCa using the TIDE algorithm and external immunotherapy datasets. Our results strongly suggest that the pathways involved in regulating *PD-L1* expression may also be considered as candidate reference factors for the comprehensive treatment of intermediate and advanced bladder tumors.

The four genes ultimately included in the PMRI were significantly and highly expressed in patients in the anti-PD-L1 immunotherapy response group. Insulin-like growth factor 2 mRNA-binding protein 3 (IGF2BP3) is an RNA-binding protein (RBP). Researchers found that IGF2BP3 can act as a *PD-L1* mRNA reader, recognizing and regulating the stability of METTL3-mediated m6A-modified *PD-L1* mRNA in a METTL3-dependent manner to prevent *PD-L1* mRNA degradation (28). The *P4HB* gene encodes a protein disulfide isomerase (PDI) and endoplasmic reticulum oxidase 1 (ERO1) that are involved in the protein folding process of the endoplasmic reticulum. Moreover, researchers found that PDI and ERO1, acting in concert, could promote oxidative protein folding of PD-L1 in the endoplasmic reticulum thereby enhancing PD-L1 expression (29, 30). Cancer cells have been reported to utilize the JAK-STAT signaling pathway to increase *PD-L1* mRNA expression, whereas *RAC3* overexpression can activate JAK/STAT signaling through the PYCR1 axis to regulate PD-L1 expression, and thus suppress tumor immunity to provide favorable conditions for BCa progression (21, 31). Similarly, the binding of Wnt ligands to activated EGFR induces β-catenin/TCF/LEF to form a complex with the PD-L1 promoter region and induces *PD-L1* mRNA expression in tumor cells. *CLK2* has been reported to increase *PD-L1* mRNA expression to evade T-cell attack via the Wnt/β-catenin/TCF/LEF pathway (32, 33). The anti-PD-L1 immunotherapy-related genes are closely related to the transcriptional, post-transcriptional, and protein stability regulation of PD-L1 in cancer cells. Consistent with these observations, we validated the function of classical PMRI genes and their relevance to PD-L1 expression in BCa cells. The results showed that IGF2BP3 promoted the proliferation and migration of BCa cells and positively regulated *PD-L1* expression. These results suggest that anti-PD-L1 immunotherapy-related genes and their derived PMRI are closely associated with PD-L1 expression and tumor immunity in cancer cells. PMRI genes from DNA induction, transcriptional, translational, and post-translational modifications of PD-L1 expression. The synergistic regulation of PD-L1 expression by PMRI genes from DNA induction, transcriptional, translational, and post-translational modifications may have potential value in therapeutic evaluation and translational research.

As another application of PITI efficacy prediction, we demonstrated the feasibility of a structure-based approach for identifying candidate small-molecule drugs that target core proteins. We used *IGF2BP3, RAC3, CLK2*, and *P4HB* as small-molecule drug targets and screened potential small-molecule drugs by molecular docking from the zinc database of Food and Drug Administration (FDA)-approved drugs using MOE software. Valrubicin is among the top four small-molecule drugs with the highest affinity for IGF2BP3, and it has been reported in clinical trials for superficial BCa chemotherapy, demonstrating that valrubicin is effective in ablating tumors remaining in the bladder after incomplete transurethral resection of bladder tumors and in preventing and delaying bladder tumor recurrence (34). A clinical trial of cobicistat, which has a strong affinity to RAC3, was conducted for the treatment of human immunodeficiency virus exposure prophylaxis (35). Irinotecan is among the top four small-molecule drugs with the highest binding affinity to the CLK2 binding pocket, and it has been widely used in clinical trials for solid tumors, such as colorectal, pancreatic, and biliary tract cancers (36–38). Furthermore, Gadofosveset, which is known to have a P4HB-docking pocket with the highest affinity, was reported in clinical trials in combination with MR angiography for the treatment of peripheral vascular disease; results showed that at 0.03 mmol/kg it was not only safe and effective for MR angiography of occlusive disease of the main iliac artery, but also had improved accuracy over non-enhanced MR angiography (39). Although the specific mechanisms of these candidate small-molecule compounds remain to be explored in depth, our findings suggest that they have great potential for anti-PD-L1 immunotherapy in BCa, especially in the population of BCa patients with high PMRI scores.

Although our constructed PMRI can closely respond to and predict the prognosis, chemotherapy sensitivity, and immunotherapy efficacy of BCa and many other cancers, this study has some limitations. First, the data for our analysis were obtained from public databases, which may have led to a case-selection bias. In addition, there is a need to collect large amounts of clinical case-data for evaluation to further validate the accuracy of our findings. Finally, further *in vivo* and *in vitro* experiments are required to validate the specific molecular mechanisms of the genes involved in the construction of the PMRI in BCa progression.

## Conclusion

In summary, based on the comprehensive analysis of multiple aspects of BCa by PMRI constructed from anti-PD-L1 immunotherapy-related genes, we found that PMRI could effectively predict the prognosis and immunotherapeutic effects in patients with BCa. This study identified novel prognoses, therapeutic biomarker combinations, and potential therapeutic targets for anti-PD-L1 immunotherapy, providing useful insights for future research on BCa treatment strategies. In the era of cancer immunotherapy, exploring co-regulation patterns of PD-L1 expression and their oncological therapeutic potential provides new perspectives for clinical diagnosis, individualized comprehensive treatment, and translational research of BCa.

## Data availability statement

The original contributions presented in the study are included in the article/**Supplementary Material**. Further inquiries can be directed to the corresponding authors.

## Ethics statement

Ethical approval was not required for the studies on humans in accordance with the local legislation and institutional requirements because only commercially available established cell lines were used.

## Author contributions

YX: formal analysis, data curation, conceptualization, experiment, writing—original draft. XYS: formal analysis, visualization, writing—original draft. GXL: software, investigation, writing—original draft. HZL: investigation, writing—original draft. MY: software, supervision, data curation, conceptualization, experiment, writing—review and editing. YYZ: conceptualization, writing—review and editing, supervision, project administration, funding acquisition. All authors contributed to the article and approved the submitted version.

## Glossary

**Table:**

ACC	Adrenocortical carcinoma
BCa	Bladder cancer
CLK2	CDC Like Kinase 2
BLCA	Bladder Urothelial Carcinoma
BRCA	Breast invasive carcinoma
CESC	Cervical squamous cell carcinoma and endocervical adenocarcinoma
CHOL	Cholangiocarcinoma
COAD	Colon adenocarcinoma
CNV	Copy number variation
CTLA4	Cytotoxic T-lymphocyte-associated protein 4
DLBC	Lymphoid Neoplasm Diffuse Large B-cell Lymphoma
DSS	Disease-free survival
ESCA	Esophageal carcinoma
GBM	Glioblastoma multiforme
GDSC	Genomics of Drug Sensitivity in Cancer
GEO	Gene Expression Omnibus
GO	Gene Ontology
GSEA	gene set enrichment analysis
GTEx	Genotype-Tissue Expression Program
HNSC	Head and Neck squamous cell carcinoma
HR	Hazard Ratio
IC50	half maximal inhibitory concentration
ICB	Immune checkpoint blockade
IGF2BP3	Insulin Like Growth Factor 2 MRNA Binding Protein 3
KEGG	Kyoto Encyclopedia of Genes and Genomes
KICH	Kidney Chromophobe
KIRC	Kidney renal clear cell carcinoma
KIRP	Kidney renal papillary cell carcinoma
KM	Kaplan–Meier
LAG3	Lymphocyte Activating 3
LAML	Acute Myeloid Leukemia
LGG	Brain Lower Grade Glioma
LIHC	Liver hepatocellular carcinoma
LUAD	Lung adenocarcinoma
LUSC	Lung squamous cell carcinoma
MESO	Mesothelioma
NES	Normalized enrichment score
OS	Overall survival
OV	Ovarian serous cystadenocarcinoma
P4HB	Prolyl 4-Hydroxylase Subunit Beta
PAAD	Pancreatic adenocarcinoma
PCPG	Pheochromocytoma and Paraganglioma
PD-1	Programmed cell death 1
PD-L1	Programmed cell death 1 ligand 1
PFS	Progression-free survival
PMRI	PD-L1 multidimensional regulatory index
PPI	protein–protein interaction
PRAD	Prostate adenocarcinoma
RAC3	Rac Family Small GTPase 3
READ	Rectum adenocarcinoma
ROC	Receiver Operating Characteristic
SARC	Sarcoma
SKCM	Skin Cutaneous Melanoma
ssGSEA	single-sample gene set enrichment analysis
STAD	Stomach adenocarcinoma
TCGA	The Cancer Genome Atlas
TGCT	Testicular Germ Cell Tumors
THCA	Thyroid carcinoma
THYM	Thymoma
TIDE	tumor immune dysfunction and exclusion
TP53	Tumor Protein P53
TMB	Tumor mutation burden
UCEC	Uterine Corpus Endometrial Carcinoma
UCS	Uterine Carcinosarcoma
UVM	Uveal Melanoma
WGCNA	Weighted Gene coexpression Network Analysis
