# Supplementary material for: Integration of multi-omics and clinical treatment data reveals bladder cancer therapeutic vulnerability gene combinations and prognostic risks
Source: Front Immunol. 2024 Jan 17;14:1301157. doi: 10.3389/fimmu.2023.1301157 (PMC10827994; doi:10.3389/fimmu.2023.1301157)
Supplement: Supplementary file 1 [file DataSheet_1.docx]

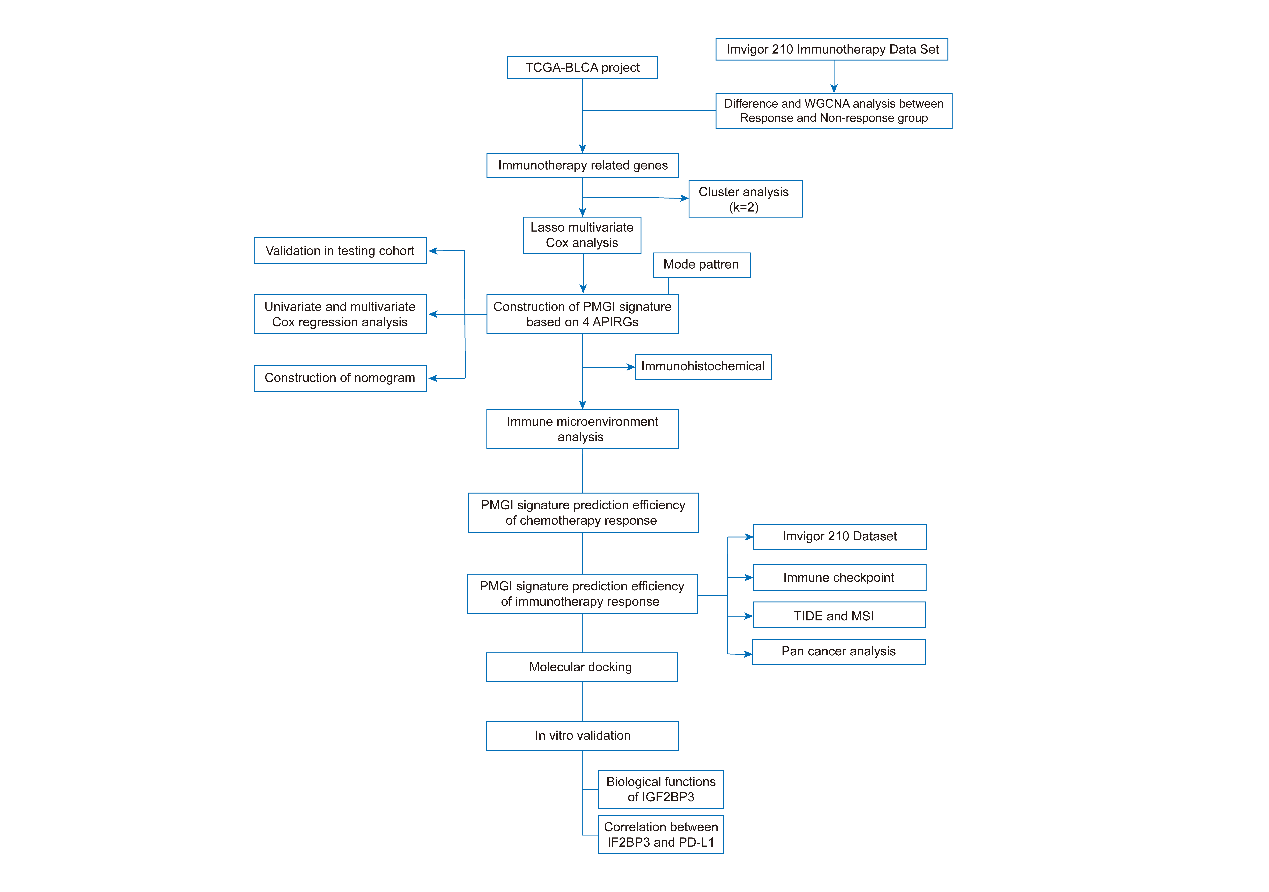


**Figure S1 Technology roadmap of this study**


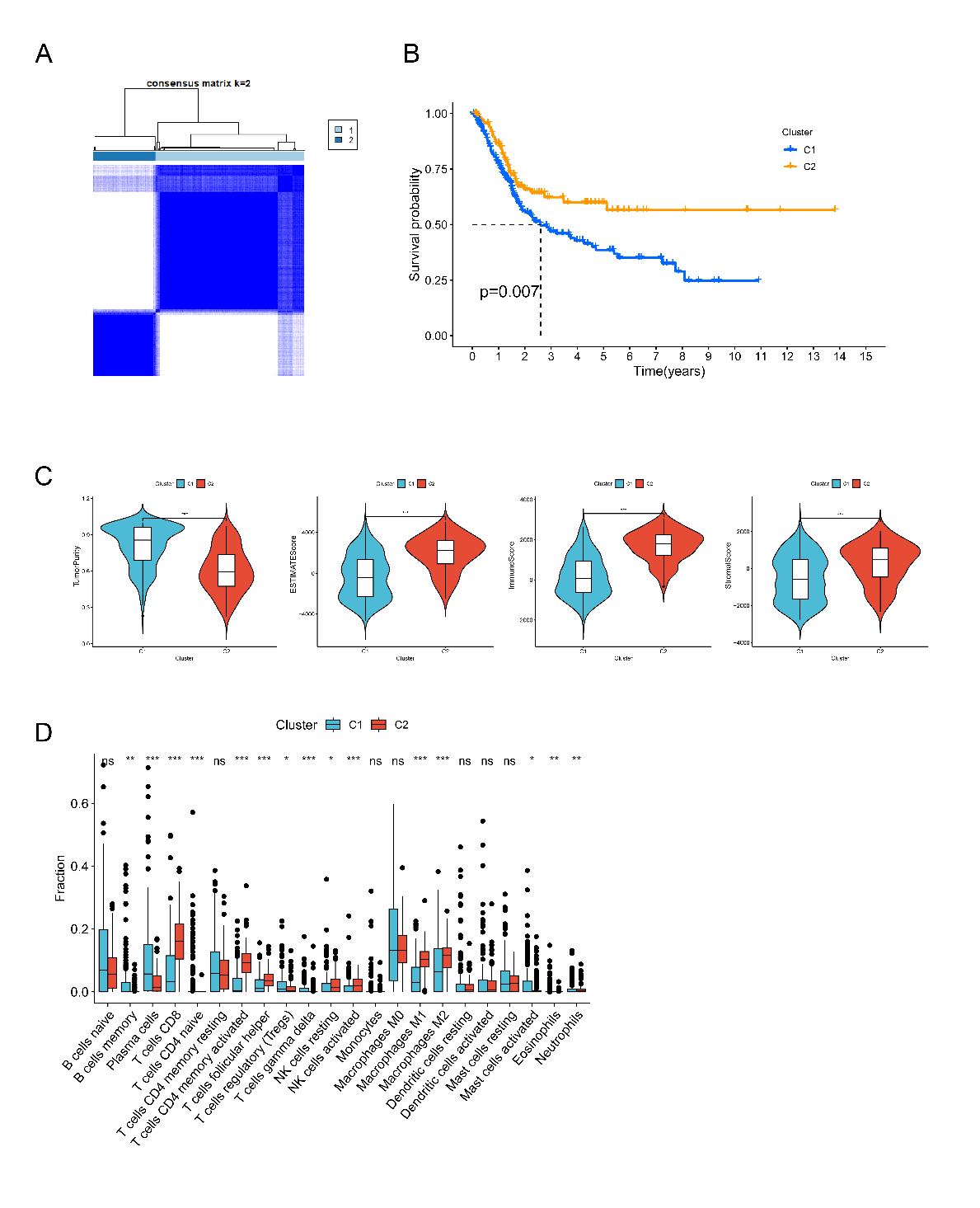


**Figure S2 Anti-PD-L1 immunotherapy clustering analysis and association between different Clusters with the immune microenvironment.** (A) Heat map of consensus clustering at k=2. (B) Comparison of Kaplan-Meier curves between Cluster 1 and Cluster 2. (C) Comparison of ESITIMATE scores of tumor purity, stroma and immunity between two Clusters. (D) Comparison of CIBERSORT scores for immune cell infiltration between the two Clusters. Note* p < 0.05, **p < 0.01, ***p < 0.001


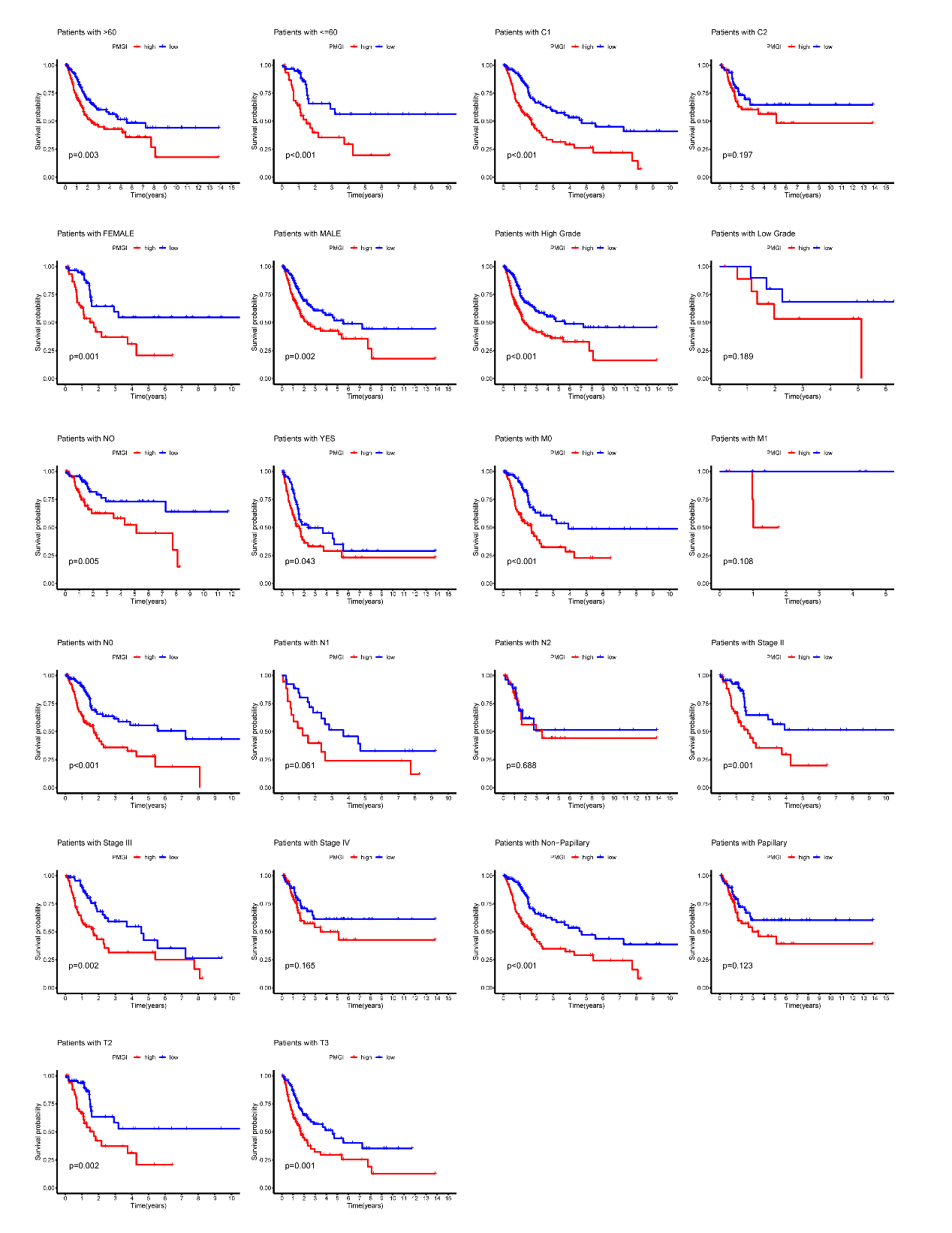


**Figure S3 Prognostic curves for clinical subgroups**


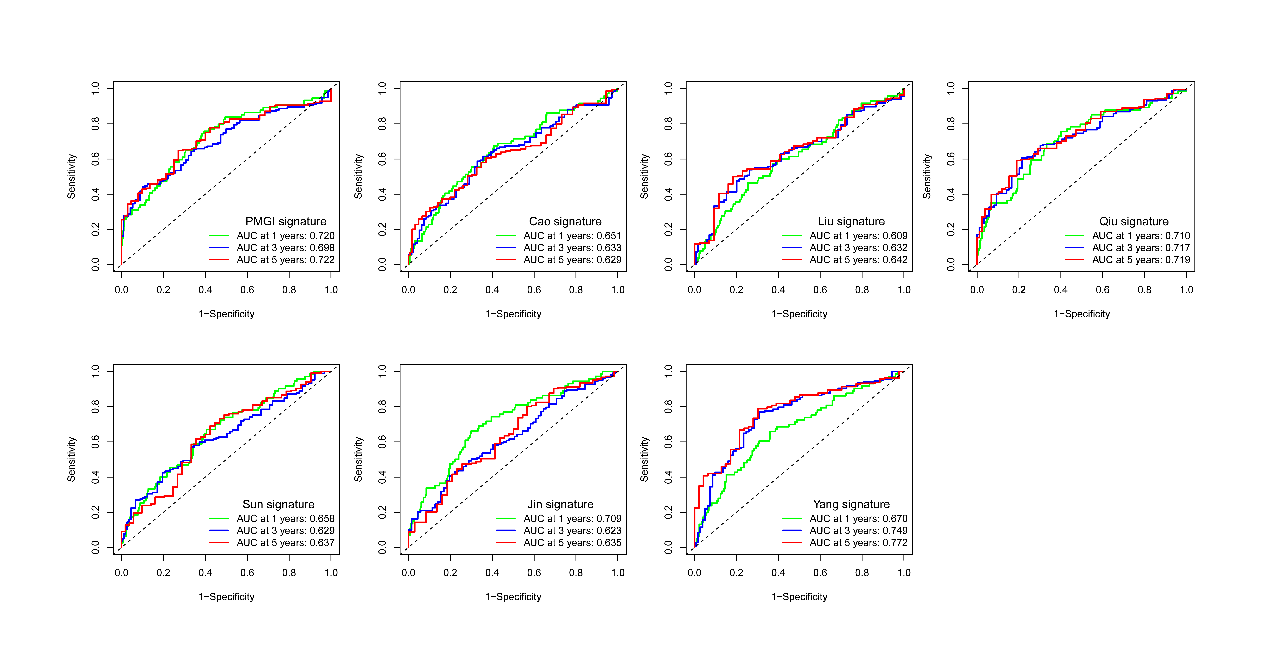


**Figure S4. Calibration curves for model comparison of ROC curves**


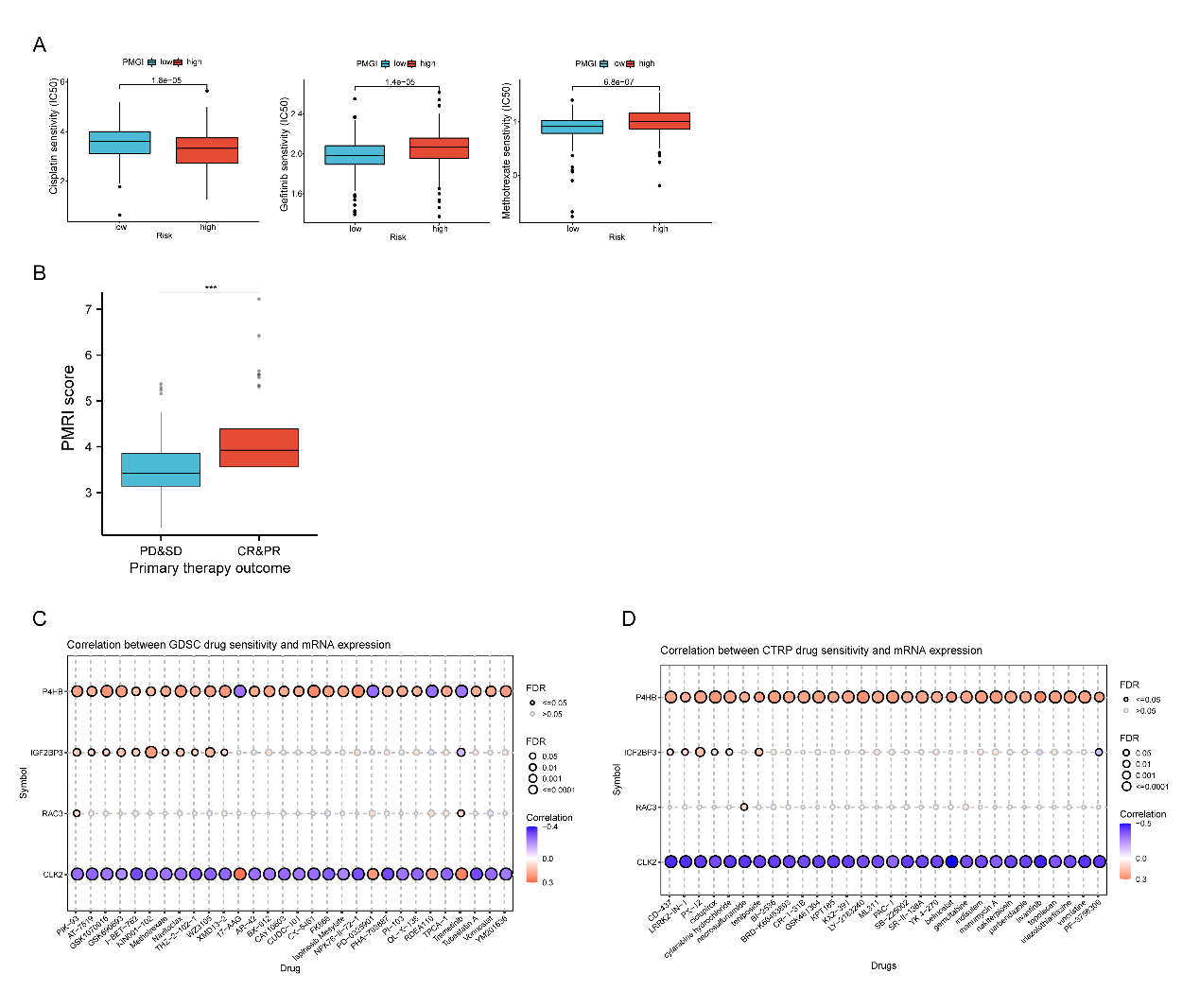


**Figure S5 Application of PD-L1 multidimensional modulation index between chemotherapy response and common drug sensitivity.** (A) Differences in response to common chemotherapy drugs between high PMGI and low PMGI groups. (B) A box chart of PMRI scores in the TCGA cohort for response to cisplatin treatment and non-response groups. Drug sensitivity and gene mRNA expression relationship were analyzed by GDSC database (C) and CTRP database (D).


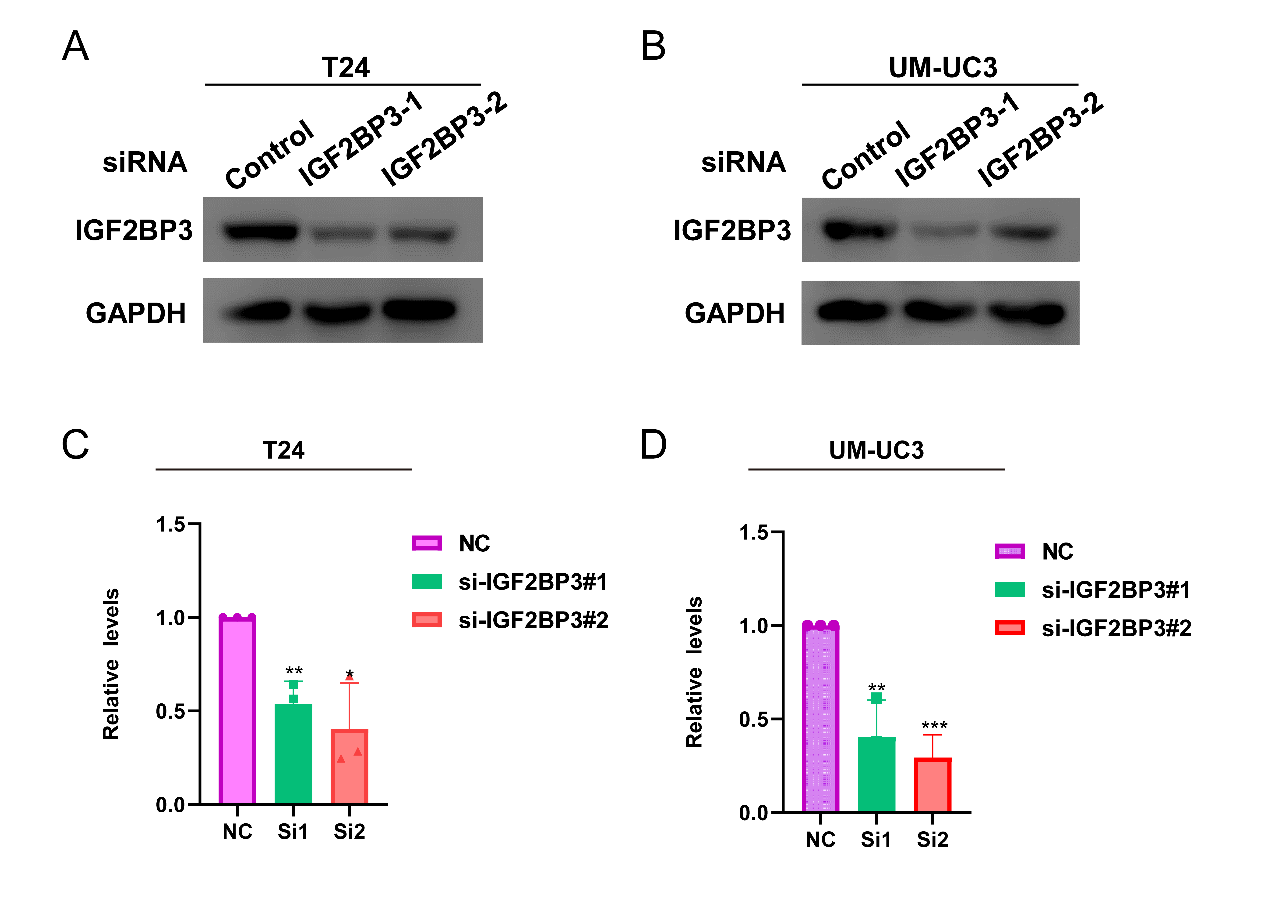


**Figure S6** (A-B) IGF2BP3 protein expression levels in T24 and UM-UC3 cells after transfection of two siRNAs targeting IGF2BP3. （C-D）Statistical histogram of PD-L1 protein expression levels in T24 and UM-UC3 cells after siRNA transfection.

**TableS1 Primer sequence**

NCBI ID 10643（IGF2BP3）

Amplicon Size 211

Sequence (5' -> 3') Length Tm Location

Forward Primer TATATCGGAAACCTCAGCGAGA 22 60.2 13-34

Reverse Primer GGACCGAGTGCTCAACTTCT 20 61.5 223-204

NCBI ID 29126(CD274)

Amplicon Size 120

Sequence (5' -> 3') Length Tm Location

Forward Primer TGGCATTTGCTGAACGCATTT 21 62 37-57

Reverse Primer TGCAGCCAGGTCTAATTGTTTT 22 60.4 156-135
